# Familiarity, orientation, and realism increase face uncanniness  by  sensitizing  to  facial distortions

**DOI:** 10.1167/jov.22.4.14

**Published:** 2022-03-28

**Authors:** Alexander Diel, Michael Lewis

**Affiliations:** 1 School of Psychology, Cardiff University, Cardiff, UK; 2 School of Psychology, Cardiff University, Cardiff, UK

**Keywords:** uncanny valley, face processing, face familiarity, face distortion, face inversion, face realism

## Abstract

The uncanny valley predicts aversive reactions toward near-humanlike entities. Greater uncanniness is elicited by distortions in realistic than unrealistic faces, possibly due to familiarity. Experiment 1 investigated how familiarity and inversion affect uncanniness of facial distortions and the ability to detect differences between the distorted variants of the same face (distortion sensitivity). Familiar or unfamiliar celebrity faces were incrementally distorted and presented either upright or inverted. Uncanniness ratings increased across the distortion levels, and were stronger for familiar and upright faces. Distortion sensitivity increased with increasing distortion difference levels, again stronger for familiar and upright faces. Experiment 2 investigated how face realism, familiarity, and face orientation interacted for the increase of uncanniness across distortions. Realism increased the increase of uncanniness across the distortion levels, further enhanced by upright orientation and familiarity. The findings show that familiarity, upright orientation, and high face realism increase the sensitivity of uncanniness, likely by increasing distortion sensitivity. Finally, a moderated linear function of face realism and deviation level could explain the uncanniness of stimuli better than a quadratic function. A re-interpretation of the uncanny valley as sensitivity toward deviations from familiarized patterns is discussed.

## Introduction

**Figure 1.**
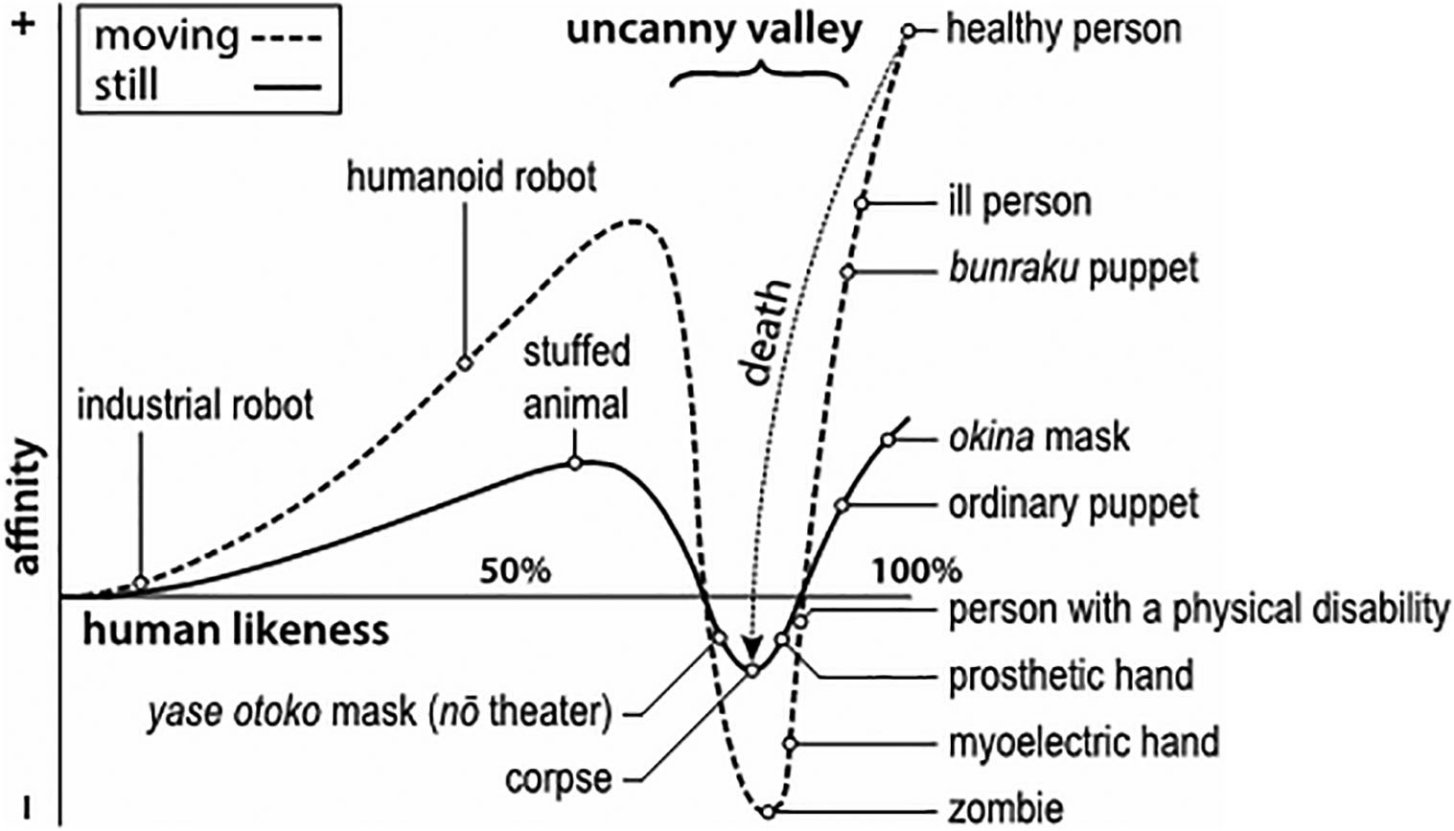
The uncanny valley as initially proposed by Masahiro Mori (courtesy to Dr. Karl MacDorman).

Artificial entities close to the human norm tend to elicit strange, cold, or eerie sensations when compared to less humanlike entities or full humans: a phenomenon called uncanny valley (UV; MacDorman & Ishiguro, 2006; Mori, 2012; see Figure 1). The UV impedes the design of humanlike artificial entities like androids or computer-generated characters with pleasant aesthetics (Pütten & Krämer, 2014; Schwind, Wolf, & Henze, 2018a). Presumably, a higher level of human likeness or realism sensitizes the aesthetic evaluations of physically distorted variants (Diel & MacDorman, 2021; Green, MacDorman, Ho, & Vasudevan, 2008; MacDorman, Green, Ho, & Koch, 2009; Mäkäräinen, Kätsyri, & Takala, 2014; Złotowski et al., 2015). As the effect hinders trust-based interaction with humanlike artificial entities (Mathur & Reichling, 2016), it remains a pressing barrier for replicating realistic robotic humans and their functional implementation into society (Ishiguro, 2007; Mende, Fischer, & Kühne, 2019). A better understanding of the mechanisms underlying the UV effect is important to overcome it in the design of humanlike entities.

### Explanations of the uncanny valley

Despite its initial connotation to artificial humanlike entities, an increase of uncanniness has also been observed for distorted or otherwise manipulated faces, and the sensitivity of aesthetic evaluations to facial deviations or exaggerations of emotional expressions increase with face realism (MacDorman et al., 2009; Mäkäräinen et al., 2014). Similarly, Green et al. (2008) found that whereas increased human likeness of a face does not decrease the range of acceptable face proportions, it does increase the sensitivity to deviations from the aesthetically best facial proportions. Thus, certain facial distortions cause uncanniness, which can be enhanced for more realistic faces.

Potential links between the UV effect and face-related processing have been suggested, like configural processing or perceptual narrowing (Almaraz, 2017; Diel & MacDorman, 2021; Kätsyri, 2018; MacDorman & Chattopadhyay, 2017). This link between the UV and face-related processing will be investigated in this paper.

### Face processing

Faces are processed in an arguably unique manner: they are detected and recognized more accurately and quicker than most other categories (Farah, Tanaka, & Draom, 1995; Richler, Cheung, & Gauthier, 2011; Valentine, 1988; Yin, 1969). Face processing has been described as feature-relational or configural and develops through experience in differentiating faces based on subtle configural cues (Rhodes, Brake, Taylor, & Tan, 1989). Global inversion disturbs a face's configuration, decreasing recognition, and discrimination ability (Carbon & Leder, 2006; Kanwisher & Moscovitch, 2000; Maurer, Le Grand, & Mondloch, 2002). Certain distortions become difficult to detect in inverted faces, like local inversions of the eyes and mouth (Thatcher illusion; Bartlett & Searcy, 1993; Lewis, 2001; Thompson, 1980). Thus, the ability to detect subtle configural deviations is reduced when the face configuration is disrupted through inversion.

The ability to discriminate individual faces is reduced for virtual faces, indicating that face expertise does not transfer to computer-generated faces (Crookes, Ewing, Gildenhuys, Kloth, Hayward, Oxner, Pond, & Rhodes, 2015). Kätsyri (2018) had participants learn and later recognize and rate a set of real and virtual faces and found a higher false alarm rate for recognizing virtual compared to real faces, again indicating difficulties in differentiating virtual faces when compared to real ones. Furthermore, inversion increased the eeriness of both virtual and real faces and more so for real ones, which Kätsyri (2018) argued to be evidence against the role of configural processing on the uncanniness of faces. However, previous research has also shown that inversion reduces the variation of aesthetic judgments of faces (Bäuml, 1994; Leder, Goller, Forster, Schlageter, & Paul, 2017; Santos & Young, 2008). Thus, configural information may instead be used to accurately assess facial aesthetics, for example, subtle configural deviations may appear less attractive or more eerie. Configural processing would then increase the range of aesthetic ratings across different face configurations due to a higher sensitivity to configural variation, including the difference between real and virtual faces. Although inversion itself may increase face eeriness in general because upside-down faces are more atypical than upright faces, inversion would then also decrease the effect of distortions on the variance of aesthetic ratings due to the decrease of perceived configural variance, and reduce the eeriness difference between real and virtual faces. Thus, it is possible that Kätysri's (2018) observation that the eeriness difference between real and virtual faces decreased when inverted may have resulted from the decreased ability to detect configural information.

Kätsyri (2018) did not manipulate the degree of face distortion, whereas the presumed moderating effect of inversion on the uncanniness of face distortions should be especially salient with a wider range of face distortions and especially notic for highly distorted faces. Specifically, inversion should lessen the increase of uncanniness across incremental facial configural distortions. In other terms, inversion should attenuate the effect of configural deviations on uncanniness by decreasing perceptual sensitivity to these deviations.

A higher level of face realism enhances sensitivity of the uncanniness of facial distortions (MacDorman et al., 2009; Mäkäräinen et al., 2014). Matsuda, Okamoto, Ida, Okanoya, and Myowa-Yamakoshi (2012) have furthermore suggested that a high degree of perceptual expertise for a face would also increase the sensitivity to deviations and fine-detail errors within the face. More generally, increased expertise or familiarity would translate into higher distortion sensitivity, and thus a stronger UV effect for humanlike compared to non-humanlike categories (e.g. distorted human compared to animal faces). Similarly, if perceptual familiarity drives the ability to detect subtle deviations, a higher distortion sensitivity and UV effect would be expected for familiar compared to novel faces. This proposal, summarized as *deviation from familiarity hypothesis*, has not yet been investigated in previous research.

As of now, only one study has tested the effect of face distortion of familiar faces on the UV effect, only with infant participants and using behavioral measures (Matsuda et al., 2012). Matsuda et al. (2012) found that infants preferred familiar (their mothers’) and unfamiliar faces over familiar-unfamiliar morphed faces. Thus, morphed faces straddling the boundaries of a familiar face are avoided by infants. However, no study has yet directly measured the effect of distortion on uncanniness for familiar and unfamiliar faces using affect ratings typical for UV research, nor for adult participants or stimulus types other than morphed faces. Similarly, previous research has not investigated how inversion affects uncanniness ratings across facial distortions. If uncanniness is mediated by the degree of expertise with a stimulus category (deviation from familiarity hypothesis), both familiarity and orientation should, however, affect uncanniness ratings across facial distortions.

### Deviation from familiarity: Understanding the uncanny valley

Investigating the effects of familiarity and orientation on distortion sensitivity should help facilitate understanding of the uncanny valley phenomenon: a more humanlike appearance activates specialized processes which link even subtle deviations from expected patterns to negative aesthetic judgments. This understanding would have different implications for prevalent theories on the UV.

A high degree of familiarity with a range of stimuli could solidify the range of acceptable variations of a configural pattern (e.g. upright faces in general or upright personally familiar faces). The distinct negative reaction of eeriness or uncanniness could then reflect a warning signal resulting from errors of predictions by violating expected, highly familiar patterns (MacDorman & Ishiguro, 2006; Saygin, Chaminade, Ishiguro, Driver, & Frith, 2012; Wang, Lilienfeld, & Rochat, 2015). In this sense, high sensitivity for deviations from familiarity may be uncanny because they violate developed expected patterns.

Some theories on the UV do not consider slight deviations to be the cause of uncanniness, and instead indicate specific markers of disease, threat, or psychopathic traits (MacDorman & Entezari, 2015; MacDorman & Ishiguro, 2006; Tinwell, Nabi, & Charlton, 2013) as evolutionary adaptive strategies. Slight deviations or changes from familiarized patterns, however, are not (necessarily) indicators of disease, and threat avoidance theories would have trouble explaining the uncanniness of slightly distorted familiar faces when the same distorted face configuration would be acceptable for an unfamiliar face. In addition, if the UV can be explained by deviations from familiarity, the effect can be expected in inorganic categories which do not pose disease-related threats to humans. Threat avoidance theories, however, predict an UV focusing on human or animal stimulus categories, not for deviations for any category given enough familiarity (see also Diel & MacDorman, 2021).

According to other theories, categorical ambiguity or categorization difficulty elicit uncanniness and thus underlie the UV (Cheetham, Suter, & Jäncke, 2011). Specifically, stimuli straddling categorical boundaries would be uncanny because of decreased processing fluency (Carr, Hofree, Sheldon, Saygin, & Winkielman, 2017; Yamada, Kawabe, & Ihaya, 2013). Alternatively, an ambiguous stimulus could be evaluated negatively due to competing categorizations of the entity, causing cognitive dissonance (MacDorman et al., 2009). Familiar faces have a narrower categorical boundary than unfamiliar faces, and inversion disrupts the categorical perception of familiar faces (Keyes, 2012). Thus, moderating effects of familiarity and inversion on the effect of distortion on uncanniness would fit theories on categorization difficulty of the uncanny valley. However, as a categorical boundary is defined as a point of qualitative shift rather than a range, an increase of distortion should not gradually increase uncanniness. Instead, uncanniness should remain constant across distortions until it drastically increases at the presumed point of distortion at which the categorization shift occurs. In addition, slightly distorted faces do not contain conflicting categorical cues and are thus unlikely to lie on a categorical boundary, and are instead better understood as deviating variants within a category. Thus, category-related theories would not predict a gradual change of uncanniness across distortions.

Some theories propose that uncanniness is caused by processes specific to the perception of humans. For example, uncanniness may result from the attribution of mind or animacy onto entities that are expected to not have these human qualities (Gray & Wegner, 2012; Stein & Ohler, 2017). Alternatively, Wang et al. (2015) proposed that uncanniness is elicited at a later stage of processing when a humanlike face is dehumanized (see also Wang, Cheong, Dilks, & Rochat, 2020). As inversion decreases the perception of mind in a face (Deska, Almaraz, & Hugenberg, 2017), an effect of inversion on uncanniness sensitivity would fit the mind attribution theory. However, neither a moderating effect of familiarity on distortion and uncanniness nor an effect of the ability to detect deviations on uncanniness are expected in these theories. Furthermore, a deviation from familiarity effect would locate the cause of uncanniness on a lower stage of processing, and expand it onto specialized stimulus categories beyond humanlike stimuli.

Finally, novelty avoidance suggest that stimulus novelty drives uncanniness (Kawabe, Sasaki, Ihaya, & Yamada, 2017; Sasaki, Ihaya, & Yamada, 2017). Because distorted familiar faces are arguably less novel than distorted unfamiliar faces given their reminiscence to familiar identities, novelty avoidance theory would not predict that distortion increases uncanniness more in familiar faces than unfamiliar faces. Alternatively, however, a sense of novelty can occur specifically because a stimulus deviates from an expected pattern or is difficult to categorize, and thus distorted familiar faces could be considered more novel than when the same distorted faces are unfamiliar.

In summary, investigating the effect of familiarity on the sensitivity to distortions has both supportive and contradicting implications for various theories on the UV. The present study aims to investigate the effect of perceptual familiarity on the uncanniness of distorted faces to test for a general cognitive mechanism of familiarity-driven deviation detection underlying the UV.

## Experiment 1

### Research question and hypotheses

Experiment 1 aimed to investigate the effect of face familiarity and inversion when interacting with the level of facial distortion, on two variables: (1) uncanniness ratings of faces, and (2) the ability to detect changes in facial distortion (*distortion sensitivity*). Previous research proposed that a high level of perceptual expertise leads to perceptions of uncanniness caused by improved detection of subtle configural distortions (e.g. Matsuda et al., 2012). Thus, uncanniness ratings should increase with increasing facial distortion (distortion main effect). This effect should be stronger for familiar (compared to novel; distortion-familiarity interaction) and upright (compared to inverted; distortion-orientation interaction) faces given the higher rate of familiarity with both familiar and upright faces.

Second, the ability to detect changes between two variants of a same face (e.g. a normal face and a slightly distorted version) should increase with a higher level of distortion difference between the faces (distortion main effect). This distortion difference level should interact with both familiarity (higher distortion sensitivity for familiar compared to novel faces) and orientation (higher distortion sensitivity for upright compared to inverted faces) if familiarity enhances the ability to detect distortions.

Finally, if uncanniness is caused by the ability to detect distortions, distortion sensitivity, here, operationalized as the degree of distortion necessary to accurately differentiate between distorted versions of the same face, should predict the sensitivity of uncanniness across different face conditions. Thus, the hypotheses are the following:

• Both face familiarity and face orientation interact with face distortion on the effect of uncanniness: familiar and upright faces show a stronger increase for uncanniness ratings with increasing the distortion levels compared to unfamiliar and inverted faces.
• Both face familiarity and face orientation interact with face distortion on the effect of distortion sensitivity: familiar and upright faces have a higher distortion sensitivity than unfamiliar and inverted faces.
• Distortion sensitivity predicts the effects of familiarity, orientation, and face distortion on uncanniness ratings.

Rating scales are the preferred method of measuring uncanniness in UV research, as they allow measuring a differentiated subjective experience (Diel, Weigelt, & MacDorman, 2022; Ho & MacDorman, 2017). For stimulus ratings, some of the most used and most effective ratings scales in uncanny valley research were used according to a meta-analysis (Diel et al., 2022): *creepy*, *eerie*, *repulsive*, and *strange*. Items were combined into an uncanniness index. In addition, human likeness was measured with a single scale. To measure distortion sensitivity, a two-back delayed face matching to sample task was used, a setup used in previous face differentiation studies (e.g. Rhodes, Hayward, & Winkler, 2006).

### Methods

#### Participants

Sixty-six participants took part in the experiment. Thirty-three British participants were recruited via the Cardiff University School of Psychology's Experimental Management System (EMS; *M_age_* = 19.15, *SD_age_* = 1.56), and 33 German participants were recruited via Prolific (*M_age_* = 24.73, *SD_age_* = 3.52). Participants either received course credits or a small monetary reward for participation.

#### Stimuli

In a preliminary study, images of 28 individuals were collected depicting frontal faces of 14 famous British and 14 famous German persons. All face stimuli were cropped to equal size, colored, and only showed the head, ears, neck, and parts of the hair. Facial expressions were either neutral or, if no neutral expression of the individual was obtainable, happy. Twenty British and 20 German participants were asked to rate whether they recognized each face and, if so, to state either the name of the person or the context the in which the person appears. The number of correct recognitions were counted for British and German participants. The five British and five German faces that were recognized most often by participants from the same country while recognized least often by participants from the other country were selected as stimuli for the main experiment. The famous British faces and the number of times recognized by the British and German participants were Philipp Schofield (33 British and 2 German), Holly Willboughly (33 British and 1 German), Anthony McPartlin (31 British and 1 German), Rylan Clark-Neal (31 British and 0 German), and Gary Lineker (21 British and 0 German). Famous German faces and the number of times recognized were Dieter Bohlen (0 British and 33 German), Thomas Gottschalk (0 British and 33 German), Stefan Raab (0 British and 32 German), Günther Jauch (0 British and 28 German), and Otto Waalkes (0 British and 26 German).

**Figure 2.**
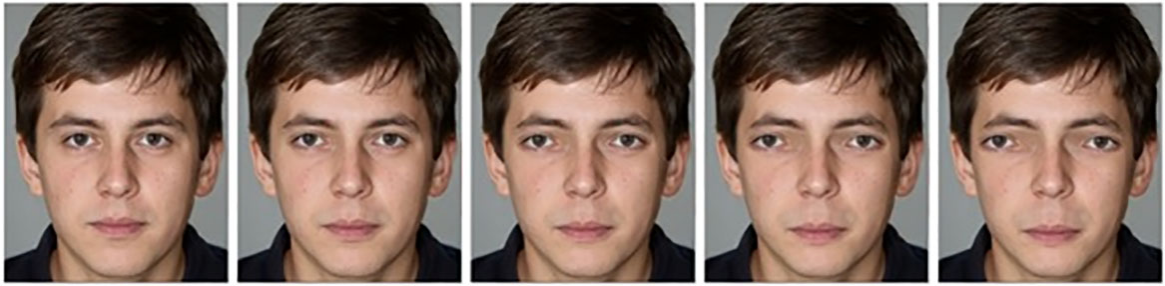
An illustration of the five examples of face stimulus distortion levels. *Note*. Faces were also presented inverted. The face depicted was not used in the experiment. The face was artificially created by the StyleGAN generative network (Karras, Laine, Aittala, Hellsten, Lehrinen, & Aila, 2020).

Photographs of those 10 selected famous persons were used as test stimuli. Each face was distorted in standardized steps by incrementally increasing the distance between the eyes while lowering the mouth. For each distortion level, interocular distance was increased by laterally displacing eyes so that the medial border of each eye's iris is placed between its original position and the position of the eye's pupil of the previous distortion level. The mouth was moved toward the chin to position the upper vermilon border between its original position and the oral fissure of the previous distortion level. Each face had five variations of incrementally increasing distortions, including the original face. Here, the term *face identity* is used to refer to an identity depicted by the face regardless of the face's distortion level, and the term *base face* to refer to the original, unedited face. Finally, all face variants were inverted on the horizontal axis to create two orientation conditions (upright and inverted). Figure 2 depicts the distortion variations one example face. Face stimuli were edited using the Photoshop CS6 software.

#### Face rating task

The first task consisted of rating each of the 100 faces (2 orientation × 5 distortion levels, for 10 face identities) on five scales: *eerie* (*unheimlich*), *creepy* (*gruselig*), *strange* (*merkwürdig*), *repulsive* (*abstoßend*), und *humanlike* (*menschenähnlich*). Each scale ranged from 0 (not at all) to 100 (fully) and were presented in the language preferred by the participant (English or German). Faces were presented randomly, and, for each face, scales were presented sequentially, simultaneously with the face. Participants had unlimited time to view the face and select a response.

#### Delayed face matching to sample task

**Figure 3.**
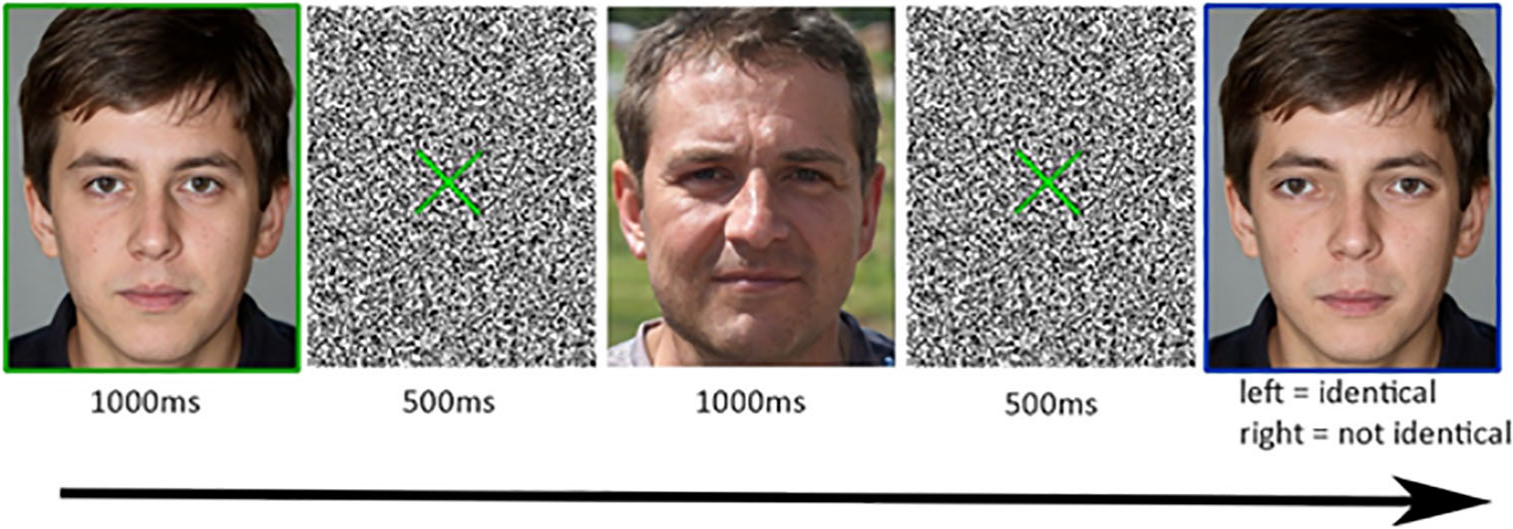
A trial in the delayed face matching to sample task. This is a mismatched trial as cue (green surround) and target (blue surround) faces are not identical. Note. The example faces were not used in the actual experiment. The faces were artificially created by the StyleGAN generative network (Karras et al., 2020).

In the second task, a cue face (surrounded by a green square) was presented followed by grey noise with a green fixation cross, a distractor (masking) face, again noise/cross, and a match face (surrounded by a blue square). Cue and match faces were always variations of the same face identity, presented in the same orientation, and were of the same or different distortion levels. Participants had unlimited time to view the match face and to decide whether the match face exactly matched the cue face. Participants had to press the left arrow key to indicate that the faces were identical, and the right arrow key to indicate that they were not identical. Masking stimuli were faces of other famous British or German persons that were not used as test stimuli in this experiment. Figure 3 depicts a single trial.

All distortion levels of a face were matched with one another, combining into 25 cue-match face pairs per face identity. Given 2 × 10 different base faces (orientation × famous person), the task consisted of a total of 500 trials where each face pair was shown once while each face appeared five times. Faces were identical 20% of the time. A break was offered every 50 trials.

#### Procedure

The study was conducted online. After receiving the link to the study, participants consented to the experiment and filled a short demographic questionnaire and a questionnaire on whether participants could recognize and identify each of the 10 famous persons. The response was used to control familiarity in the experiments. Participants then completed the face rating task first and the delayed face matching to sample task second. Because the exposure to each face was higher in the matching compared to the rating task, the rating task was conducted first to reduce the effect of familiarization on the experiments. After the study, participants received a debriefing.

#### Statistical analysis

For the first hypothesis, a 2 × 2 × 5 (orientation × familiarity × distortion level) analysis was conducted for uncanniness ratings, with orientation, familiarity, and distortion level as fixed effects and face identities and participants as random effects. For the second hypothesis, a 2 × 2 × 5 (orientation × familiarity × distortion difference level) analysis was conducted for “identical” response rate, with orientation, familiarity, and distortion difference level as fixed effects and face identities as random effects. For the third hypothesis, “identical” response rates were added as a fixed-effect predictor for the model used for hypothesis 1. Data cleaning was conducted by removing all interquartile range outliers for each distortion condition (distortion levels 0 to 4). Data preparation, data cleaning, and statistical analyses were conducted in R software. Linear mixed models were used for hypotheses 1 to 3 because they allow to deal with both fixed effects and random effects (McLean, Sanders, & Stroup, 1991), which are expected in the present study given the within-subject and within-face design. Linear mixed models are more appropriate than standard ANOVA here because of the need to control for the effect of face identity. This type of analysis produces the large degrees of freedom that can be observed below (see also Kuznetsova, Brockhoff, & Christensen, 2017; Luke, 2017). The R software packages *lme4* (for linear mixed models, using the function *lmer()*) and *lmerTest* (for complete depiction of the results), and *robustlmm* were used (Bates, Mächler, Bolker, & Walker, 2015).

#### Ethics statement and data availability

The study was approved by the Cardiff University School of Psychology Research Ethics Committee in in November 2020 (reference number: EC.20.10.13.6081GR). The data and the R code for the analysis are available at: https://osf.io/7prax/?view_only=f4583a5e9d5541778341a632f40499d2.

### Results

#### Task 1: Face rating task

##### Scale evaluation

The scales eerie, creepy, strange, and repulsive were combined into a single uncanniness index by calculating the mean values across the four scales after correcting scale inversions. The index’ Cronbach's alpha was *α* = 0.94, indicating strong reliability. The correlation matrix of the scales is depicted in Table 1.

**Table 1.** Intercorrelations between the scales used for the uncanniness index (α = 0.94).

	Eerie	Creepy	Strange	Repulsive
Eerie	1			
Creepy	0.87	1		
Strange	0.80	0.80	1	
Repulsive	0.78	0.80	0.76	1

##### Uncanniness and human likeness

**Figure 4.**
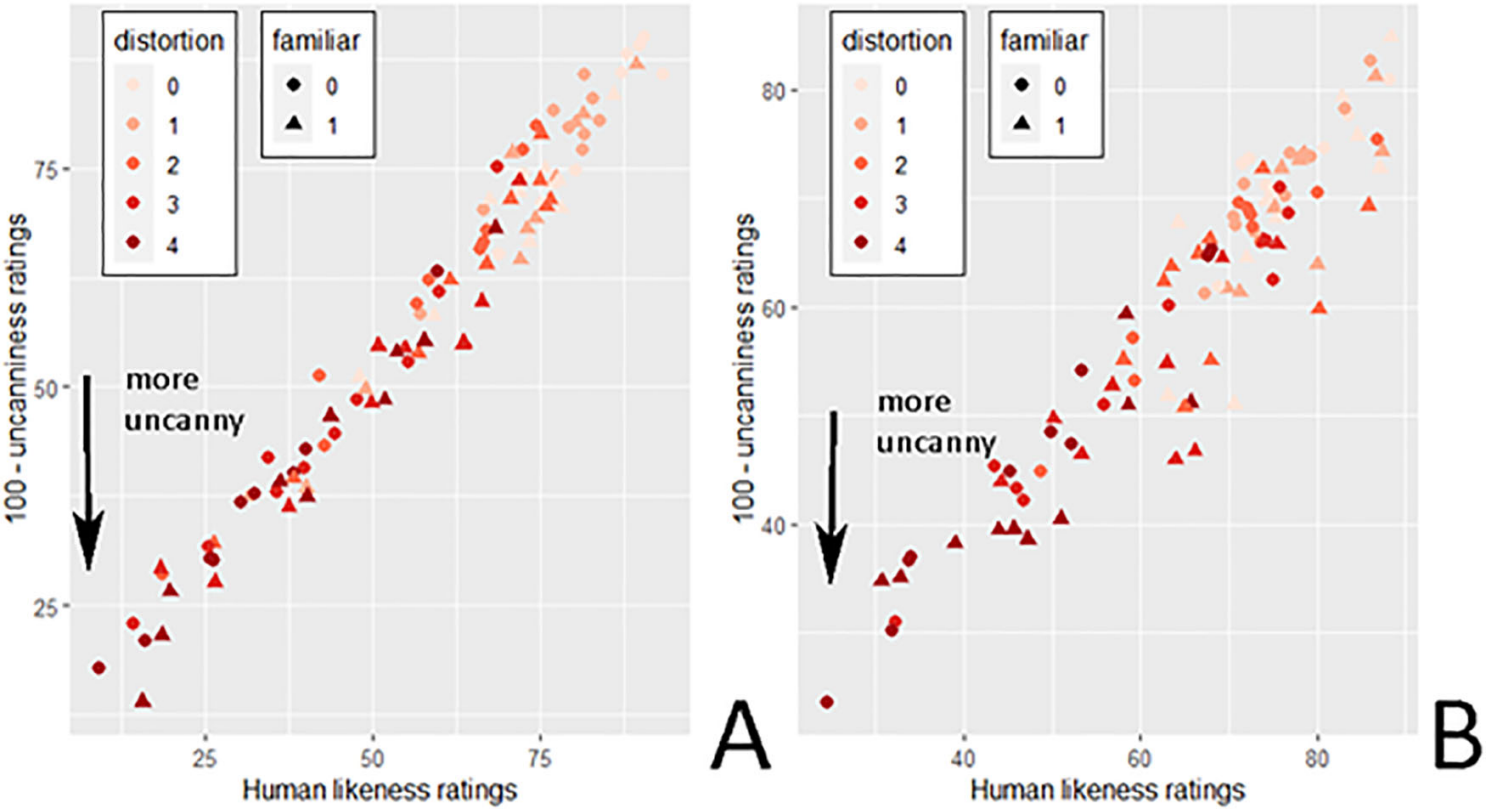
Uncanniness ratings as a function of human likeness ratings for (**A**) upright and (**B**) inverted faces, across distortions levels (0 = base face) and face familiarity. The “100- uncanniness ratings” represent the y-axis of Mori's (2012) original uncanny valley curve, with lower values depicting higher uncanniness ratings.

Uncanniness ratings were plotted as a function of human likeness. A linear mixed model could explain the distribution (*t*(502) = −81.22, *p* < 0.001), whereas a quadratic model could not (*t*(5108) = −3.021, *p* = 0.239). Thus, the relationship between uncanniness and human likeness is best explained by a linear function. Figure 4 shows a scatterplot with each point depicting a trial, for both upright and inverted faces (note the inverted scale to map the data to the typical UV plot in Figure 1).

Post hoc linear mixed model analyses found that human likeness ratings decreased with increasing distortion levels (*t*(5037) = −29.551, *p* < 0.001) and that novel faces were more humanlike than familiar faces (*t*(5055) = 3.24, *p* = 0.001). However, no main effect of orientation was observed (*t*(18) = 0.871, *p* = 0.395). Distortion interacted with both familiarity (*t*(5037) = 5.678, *p* < 0.001) and orientation (*t*(5037) = 10.194, *p* < 0.001).

##### Prediction of uncanniness

**Figure 5.**
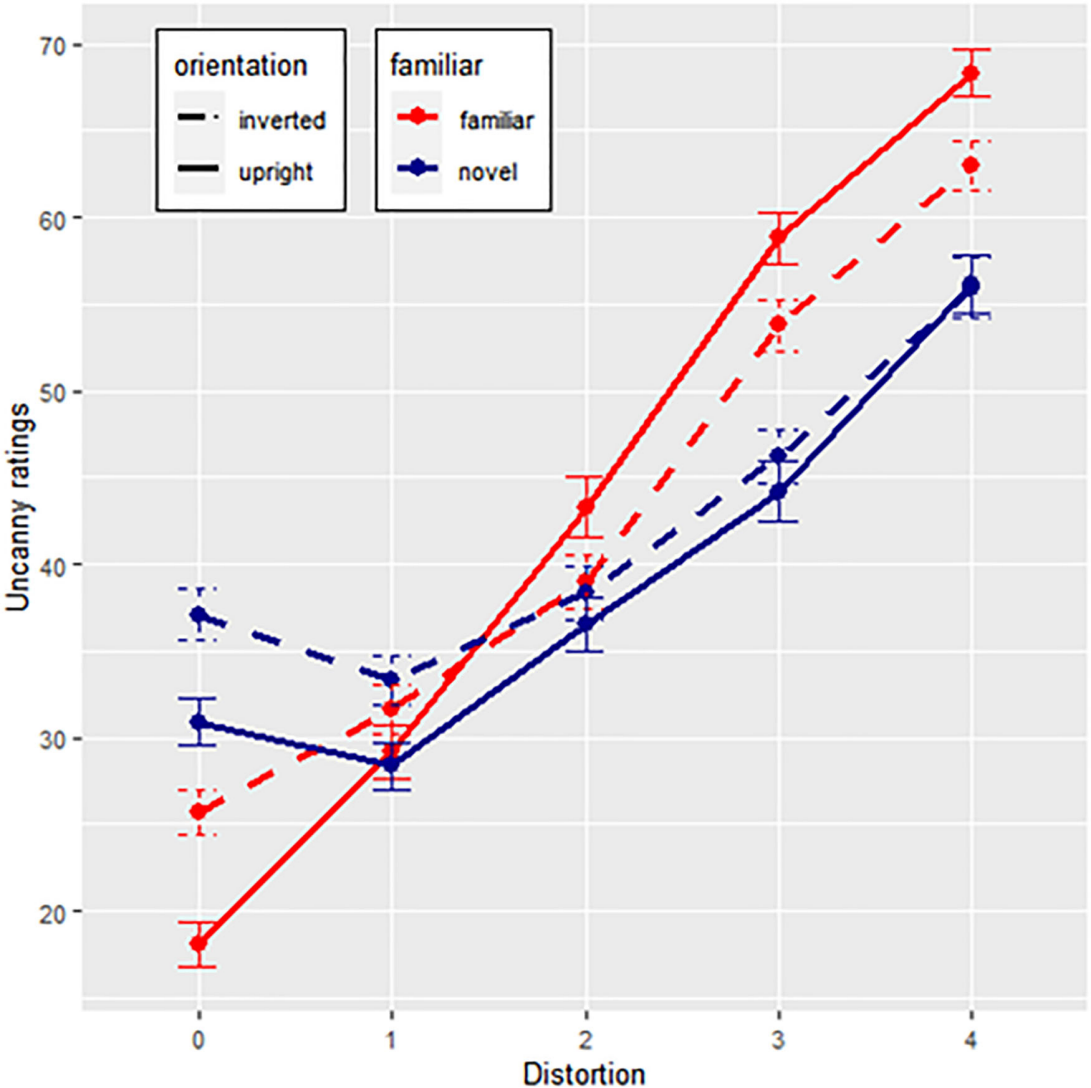
Uncanny ratings across face distortion levels (0 = original face, 4 = most distorted face). Red and blue lines depict ratings for familiar and unfamiliar faces, whereas slashed and full lines depict response rates for inverted or upright faces. Error bars show +/−1 standard errors based on within-subject variability.

A summary of uncanniness ratings across conditions is depicted in Figure 5. Orientation, familiarity, and distortion were used as fixed effects to predict uncanniness, and face identity and participants as random effects. As the assumption of homoscedasticity was not met, a robust estimation of the linear mixed model was calculated. Distortion significantly predicted uncanniness (*t*(6308) = 32.483, *p* < 0.001), but neither familiarity (*t*(6317) = 0.257, *p* = 0.798) nor orientation (*t*(19) = −1.073, *p* = 0.297). Interaction effects between distortion and familiarity (*t*(6308) = −6.204, *p* < 0.001), distortion and orientation (*t*(6308) = −11.573, *p* < 0.001), and familiarity and orientation (*t*(6321) = 2.644, *p* = 0.008) were found, as well as an interaction with all factors combined (*t*(6308) = 2.588, *p* = 0.010). The model's regression coefficient was *R²_corr_* = 0.458. The test statistics for all terms are summarized in Table A1. Data are summarized in Figure 5.

Post hoc Tukey tests with Bonferroni corrections were performed to test differences between face condition groups (familiar upright versus novel upright, familiar upright versus familiar inverted, novel upright versus novel inverted, and familiar inverted versus novel inverted) for each distortion level. At distortion level 0, novel upright faces were more uncanny than familiar upright faces (*t*(65) = 4.657, *p_adj_* < 0.001), familiar inverted faces were more uncanny than familiar upright faces (*t*(65) = 6.324, *p_adj_* < 0.001), and novel inverted faces more uncanny than familiar inverted faces (*t*(65) = 2.748, *p_adj_* = 0.031) and novel upright faces (*t*(65) = 5.103, *p_adj_* = < 0.001). Thus, both novelty and inverted orientation increased uncanniness of base faces. At distortion level 1, no differences between condition groups were significant. At distortion level 2, all differences were nonsignificant except for familiar inverted faces, which were less uncanny than familiar upright faces (*t*(65) = −4.482), *p_adj_* = < 0.001). Thus, at distortion level 2, upright orientation increased uncanniness ratings for familiar faces. Familiar inverted faces remain less uncanny than familiar upright faces at distortion level 3 (*t*(65) = −8.47, *p_adj_* < 0.001), and novel inverted faces become less uncanny than familiar inverted faces (*t*(65) = −4.331, *p_adj_* < 0.001). Thus, at this stage, inversion generally reduces the uncanniness of distorted faces. Finally, at distortion level 4, familiar inverted faces again remain less uncanny than familiar upright (*t*(65) = −8.072, *p_adj_* < 0.001), and novel inverted faces less uncanny than familiar inverted faces (*t*(65) = −4.727, *p_adj_* < 0.001). In addition, novel upright faces are less uncanny than normal familiar faces (*t*(65) = −2.963, *p_adj_* = 0.023), suggesting that both upright orientation and familiarity increase the uncanniness of distorted faces. These results show that uncanniness increases the strongest across distortion levels when faces are upright (versus inverted) and familiar (versus novel). Thus, hypothesis 1 was supported.

#### Task 2: Face matching task

##### Prediction of “identical” response rates

**Figure 6.**
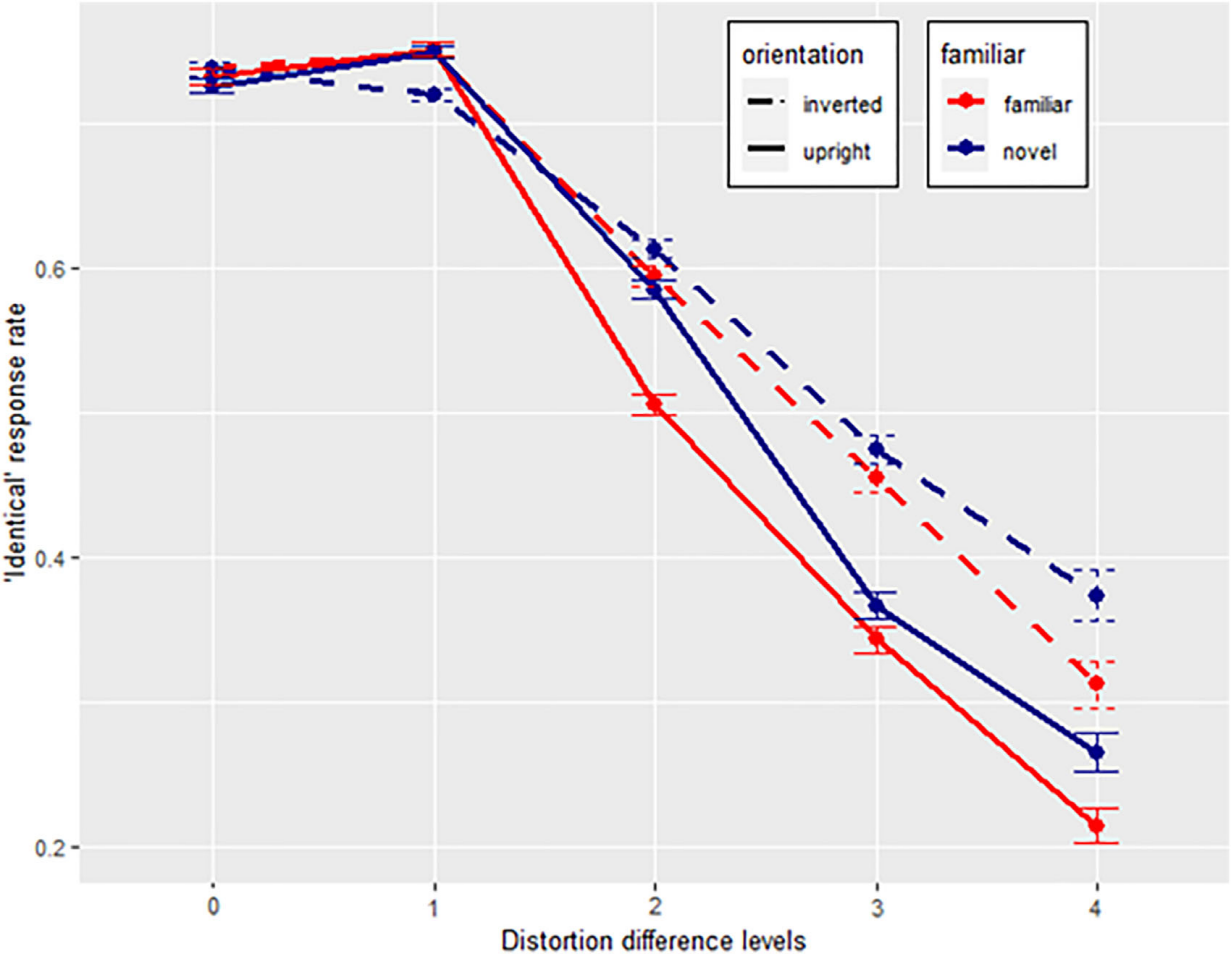
Identical response rates across face distortion difference levels (0 = cue and match face were identical, 4 = cue and match face were 4 distortion levels apart) Red and blue lines depict familiar and unfamiliar faces, whereas dashed and full lines depict response rates for inverted and upright faces. Error bars show +/−1 standard errors based on within-subject variability. Note. Distribution bars represent standard deviations.

**Figure A1.**
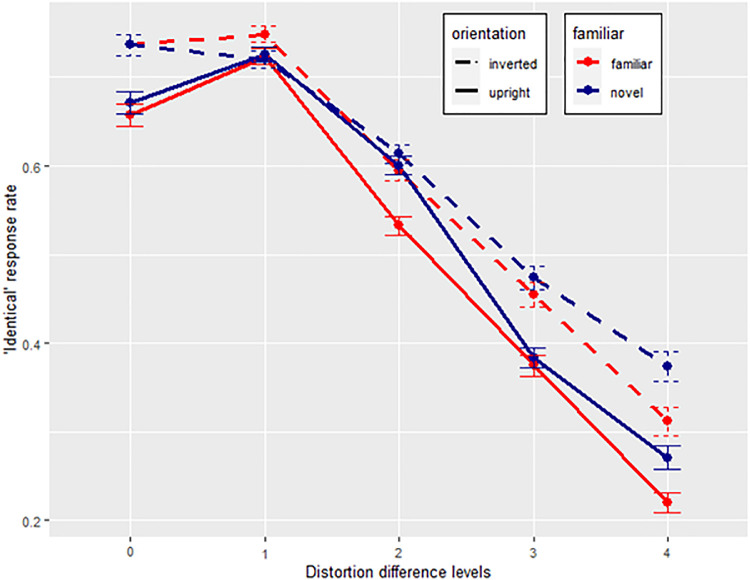
Identical response rates across face distortion difference levels (0 = cue and match face were identical, 4 = cue and match face were 4 distortion levels apart). Red and blue lines depict familiar and unfamiliar faces, whereas dashed and full lines depict response rates for inverted and upright faces. Error bars show +/−1 standard errors based on within-subject variability. Note. Distribution bars represent standard deviations.

All participants with an “identical” response rate of equal or less than 25 between distortion difference levels 0 and 4 were excluded, as no difference in response behavior between the end point distortion levels indicates that participants answered at random. A total of 16 data sets were excluded from the response rate analysis, leaving *n* = 50 participants (21 British and 29 German). Data are summarized in Figure 6. A plot which includes only those trials with at least one base face is seen in Figure A1.

Orientation, familiarity, and distortion difference level were included as fixed effects to predict identical response rates, and face identity, and participant as random effects. The assumption of homoscedasticity was not met, hence, a robust estimation of the linear mixed model was performed. Distortion difference levels (*t*(24900) = −65.097, *p* < 0.001), familiarity (*t*(24910) = 10.996, *p* < 0.001), and orientation (*t*(24370) = 16.853, *p* < 0.001) all significantly predicted identical response rates, just as interactions between distortion and familiarity (*t*(24900) = 5.419, *p* < 0.001), distortion and orientation (*t*(24900) = 10.707, *p* < 0.001), and familiarity and orientation (*t*(24910) = −4.989, *p* < 0.001). The model's regression coefficient is *R²_corr_* = 0.449.

Post hoc Tukey tests were conducted to test differences between condition groups (familiar upright versus familiar inverted, familiar upright versus novel upright, novel upright versus novel inverted, and familiar inverted versus novel inverted) across distortion difference levels. At distortion difference levels 0 and 1, no tested differences were significant. At distortion difference level 2, only familiar inverted faces had a higher identical response rate than familiar upright faces (*t*(41) = 3.559, *p_adj_* = 0.007). Thus, at distortion difference level 2, familiar faces were easier to discriminate when they were upright compared to inverted. At distortion difference level 3, familiar inverted faces remained more difficult to differentiate than familiar upright faces (*t*(65) = 3.618, *p_adj_* = 0.006), in addition to novel inverted faces having a higher identical response rate than novel upright faces (*t*(65) = 3.441, *p_adj_* = 0.01). Thus, inversion decreased the general ability to differentiate between faces at this distortion difference level. Finally, at distortion difference level 4, familiar inverted faces had still a higher identical response rate than familiar upright faces (*t*(65) = 3.39, *p_adj_* = 0.006) and novel inverted faces higher than novel upright faces (*t*(65) = 3.441, *p_adj_* = 0.01). In addition, novel inverted faces had a higher identical response rate than familiar inverted faces (*t*(65) = 2.206, *p_adj_* = 0.016), but the difference between familiar and novel upright faces remained nonsignificant. Identical response rate decreased stronger for upright faces across distortion levels than for inverted faces, especially when faces were familiar. Thus, hypothesis 2 is supported.

#### Distortion sensitivity as a predictor of uncanniness

According to the third hypothesis, the ability to detect distortion differences of the same face can explain the effects of familiarity, orientation, and distortion on uncanniness. Thus, the rate of identical responses (study 2) was added to the prediction model of uncanniness (study 1). Because the variables from the two studies were coded differently (uncanniness ratings are linked to individual stimuli, whereas response rates are linked to pairs of two stimuli), only study 2 trials with a base face as either cue or target face were included, and response rates were linked to the uncanniness ratings of the face paired with the base face (or of the base face if cue and target were identical).

A linear mixed model was calculated either with identical response rate, or familiarity, orientation, and distortion as fixed effects and face identity and participants as random effects. Because the assumption of homoscedasticity was not met, robust estimations were calculated. Significant main effects for all predictors (for familiarity *t*(9388) = 6.684, *p* < 0.001; for orientation *t*(9120) = −11.077, *p* < 0.001; for distortion *t*(9386) = 34.314, *p* < 0.001; and for response rate *t*(9340) = 2.232, *p* = 0.026) were found. Furthermore (and in correspondence to the previous regression analyses), the interactions between familiarity and orientation (*t*(9385) = 7.029, *p* < 0.001), distortion and familiarity (*t*(9380) = −4.819, *p* < 0.001), distortion and orientation (*t*(9281) =−11.051), *p* < 0.001), and distortion, familiarity, and orientation combined were significant (*t*(9378) = 3.702, *p* < 0.001). The interactions remain significant when adding the identical response rate as a predictor (for familiarity, orientation, and response rate *t*(9382) = 2.188, *p* = 0.029; for distortion, familiarity, and response rate *t*(9381) = −5.736, *p* < 0.001; for distortion, orientation, and response rate *t*(9382) = −6.900, *p* < 0.001; and for all predictors combined *t*(9379) = 3.348, *p* < 0.001). The model's regression coefficient is *R²_corr_* = 0.511.

A model with response rate alone could predict uncanniness ratings (*t*(9431) = −38.37, *p* < 0.001, *R²* = 0.371). The three factors of orientation, familiarity, and distortion could predict the response rate, with an *R²* of 0.451. Thus, hypothesis 3 was supported.

### Discussion

#### Human likeness ratings

The results show a linear relationship between human likeness and uncanniness. As realistic faces and their distortions were used in this study and no less humanlike stimuli, the results are not surprising: the stimulus range and data likely reflect the rightmost part of the valley or the range from the low point of the valley to full human likeness. Post hoc analyses found interaction effects between the distortion level and the face orientation and familiarity. Specifically, human likeness decreased stronger with increasing distortion levels when faces were upright (versus inverted) and familiar (versus novel). These findings reflect those of uncanniness ratings: upright orientation and familiarity increase the sensitivity to human likeness perception caused by configural deviations from “normal” faces. Hence, the findings suggest that a disruption of the configural, upright face pattern also disrupts the accuracy of human likeness ratings similar to the perception of humanness in inverted faces found in previous research (Hugenberg, Young, Rydell, Almaraz, Stanko, See, & Wilson, 2016).

However, an increase of uncanniness along a manipulation variable alone is not sufficient to locate a stimulus range across a “proper” UV curve because the range of human likeness to the left of the observed data is missing. Thus, additional research is needed to investigate the association between face distortion and an UV plot.

#### Familiarity, orientation, and uncanniness

Results show significant interactions among distortion levels, familiarity, and orientation of faces on uncanniness. Uncanniness increased across distortion levels, and this effect was reduced when faces were inverted while familiarity enhances the effect. Results thus support hypothesis 1.

#### Familiarity, orientation, and distortion sensitivity

In tune with hypothesis 2, familiarity and upright orientation increases the distortion sensitivity of faces. Results show significant interactions between familiarity and distortion difference and orientation and distortion difference. Specifically, both familiarity and an upright orientation increased participants’ abilities to differentiates variants of the same face.

#### Distortion sensitivity as a mediator for uncanniness

In accordance with previous research, stimulus categories participants are expectedly more familiar with are more sensitive to uncanniness when distorted (Chattopadhyay & MacDorman, 2016; Diel & MacDorman, 2021; MacDorman et al., 2009; Mäkäräinen et al., 2014; Matsuda et al., 2012). Perceptual experience or familiarity could affect uncanniness by increasing the viewer's ability to detect subtle configural differences of a stimulus, thus increasing the likelihood to detect subtle deviations which are then perceived as uncanny. Although face inversion would reduce this ability because of the specialization for upright faces, familiarity would in turn enhance it. This study's results found that the response rate alone could predict uncanniness. Thus, distortion sensitivity may in fact mediate the effect of familiarity and orientation of the sensitivity to uncanniness across distortions.

## Experiment 2

Although Experiment 1 found that the sensitivity to uncanniness is stronger for upright and familiar faces, the results do not allow an interpretation in the context of the UV. Whereas it is possible that the range of stimuli encompasses the rightmost part of the UV curve, this relationship has not been tested here. Furthermore, it is unclear how the degree of realism interacts with the observed effects on uncanniness sensitivity. Thus, Experiment 2 was designed to investigate whether the faces observed in Experiment 1 can be placed within a “proper” UV function, and how the level of realism interacts with familiarity and upright orientation.

### Research question and hypotheses

Previous research suggests that facial distortions are more acceptable for less realistic faces (e.g. Mäkäräinen et al., 2014). This has anecdotal face value as cartoon characters are liked despite exaggerated, stylized proportions of a face or facial features which would be unacceptable for more realistic faces. One explanation is that a higher level of realism directly increases the sensitivity to deviations by decreasing the range of acceptable variation of facial structure. According to the face space framework (Valentine, 1991; Valentine, Lewis, & Hills, 2016), human faces can vary on different dimensions of facial structure. Normal variations on these dimensions which are typically observed in everyday life would create an experience-based, “acceptable” range of facial structure, whereas exaggerated values on these face space's dimensions would lead to unusual, distorted faces places beyond this acceptable or normal range. Less realistic faces could miss important details that allow the estimation of the face's structure, which would decrease the ability to detect deviating variations and thus increase the range of acceptable face structures. Furthermore, the effect of lower realism on acceptable face variations would be more increased for inverted faces as inversion has been shown to decrease distortion sensitivity in study 1. However, face familiarity should curb the effect of low face realism on distortion, as a distorted familiar face would be judged more harshly based on its difference from the familiar norm rather than the general face norm.

Thus, the following hypotheses are proposed:

1. Uncanniness of faces ranging on distortion, familiarity, orientation, and realism produce an uncanny valley-like, quadratic function when plotted against their human likeness.
2. Familiarity, upright orientation, and high face realism increase the effect of distortion on uncanniness. Specifically, the increase of uncanniness across distortions is higher in more realistic faces than low realistic faces, and more so for familiar (versus novel), and upright (versus inverted) faces*.*

### Methods

#### Participants

Forty-two participants have been UK participants recruited via Prolific. Participants’ age was *M_age_* = 24.58, *SD_age_* = 4.93, and 67.5% were women.

#### Stimuli

**Figure 7.**
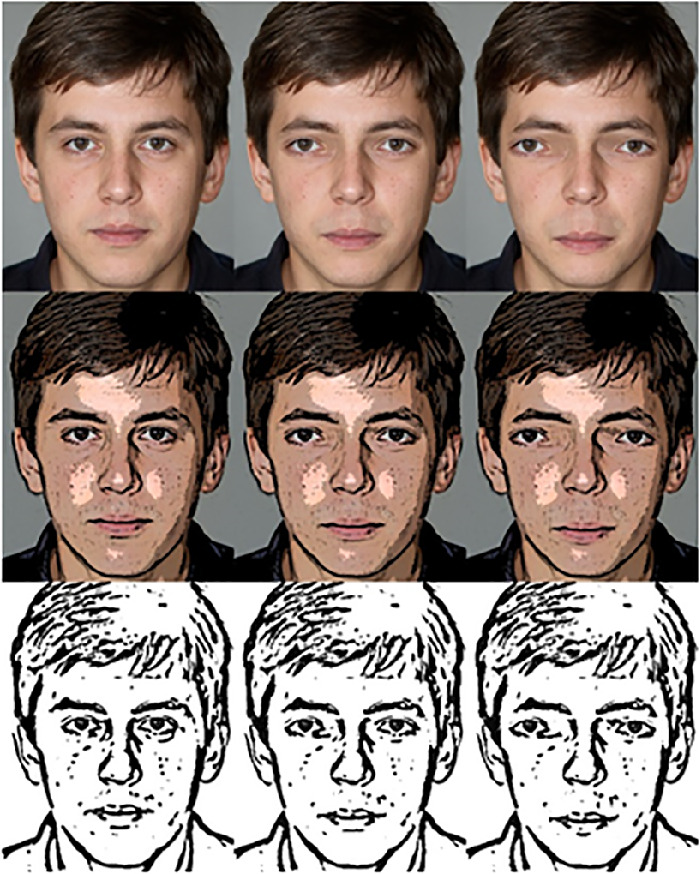
Example stimuli across distortion levels (0, 2, and 4; left to right) and realism levels (real, block print style, drawing style; and up to down). Note. Depicted example faces were not used in the actual experiment.

Stimuli were selected and created to vary along familiarity (familiar versus upright), orientation (upright versus inverted), face distortion level (3 levels), and realism level (3 levels). First, all stimuli from study 1 with the distortion levels 0 (base face), 2, and 4 were again used in this experiment. Only three distortion levels were used to limit the total number of stimuli. In addition, two low realism (stylized) variants of each used famous face were created using the following methods: (1) block print style, created by adding a poster edges filter to the faces in Photoshop CS6, and (2) drawing style, created by adding using the smart blur, high pass, threshold, and palette knife tools in Photoshop CS6. Image manipulation was loosely based on the method used in Mäkäräinen et al. (2014). Images consisted of five (realism) times three (distortion) times two (familiarity) times two (orientation) times five (exemplars), adding up to a total of 300 stimuli. Examples of the stylization across distortion levels are seen in Figure 7.

Because these stylized versions of famous faces can themselves be considered deviations from familiar faces, a total of 20 (2 familiarity conditions × 2 realism levels × 5 faces) faces of real cartoon characters were additionally selected and analogously distorted on three distortion levels. To control face familiarity, faces were either internationally famous or from Soviet or Russian cartoons. Furthermore, faces were either 2D- or 3D-animated to control for the level of detail. Five famous 2D animated faces were of Mickey Mouse (Disney), Homer Simpson (The Simpsons), Shaggy (Scooby Doo), Fred Flintstone (The Flintstones), and Stewie Griffin (Family Guy). Five Soviet/Russian 2D animated faces were Uncle Fyodor (Three from Prostokvashino), Malish (Soviet animated version of Karlson from the roof), Ivan Zarevich (Ivan Zarevich and the Grey Wolf), Alyosha Popovich (Three Bogatyrs), and Jim Hawkins (Soviet animated version of Treasure Island). Five famous 3D animated faces were Super Mario (Nintendo), Elsa (Disney's Frozen), Buzz Lightyear (Disney's Toy Story), Wallace (Wallace and Grommit), and Shrek (Shrek). Soviet/Russian 3D animated faces were Masha (Masha and the Bear), Cheburashka (Cheburashka/Gena the Crocodile), the smallest gnome (samyy malenkiy gnom), Dim Dimych (Fixiki), and Boria/Valery (Fantasy Patrol). Soviet or Russian animated characters were selected because of the wide range of animated series available mostly unknown to Western audiences. All faces were either upright or inverted, creating a total of 300 faces (2 familiarity × 2 orientation × 5 realism levels × 3 distortion level × 5 faces). Selecting images of different characters or objects is one of the most common practices in uncanny valley research (see *distinct entities* in Diel et al., 2022).

#### Procedure

After giving informed consent, participants completed a short demographic questionnaire and followed a link to the face rating task. The face rating task was identical to the face rating task in Experiment 1.

### Results

#### Rating scales

The scales *eerie*, *creepy*, *strange*, and *repulsive* were combined to a single *uncanniness* index. The index’ Cronbach's alpha was *α* = 0.93, indicating strong reliability. Inter-scale correlations are summarized in Table 2.

**Table 2.** Intercorrelations between the scales used for the uncanniness index.

	Eerie	Creepy	Strange	Repulsive
Eerie	1			
Creepy	0.85	1		
Strange	0.76	0.80	1	
Repulsive	0.73	0.77	0.67	1

#### Uncanny valley

**Figure 8.**
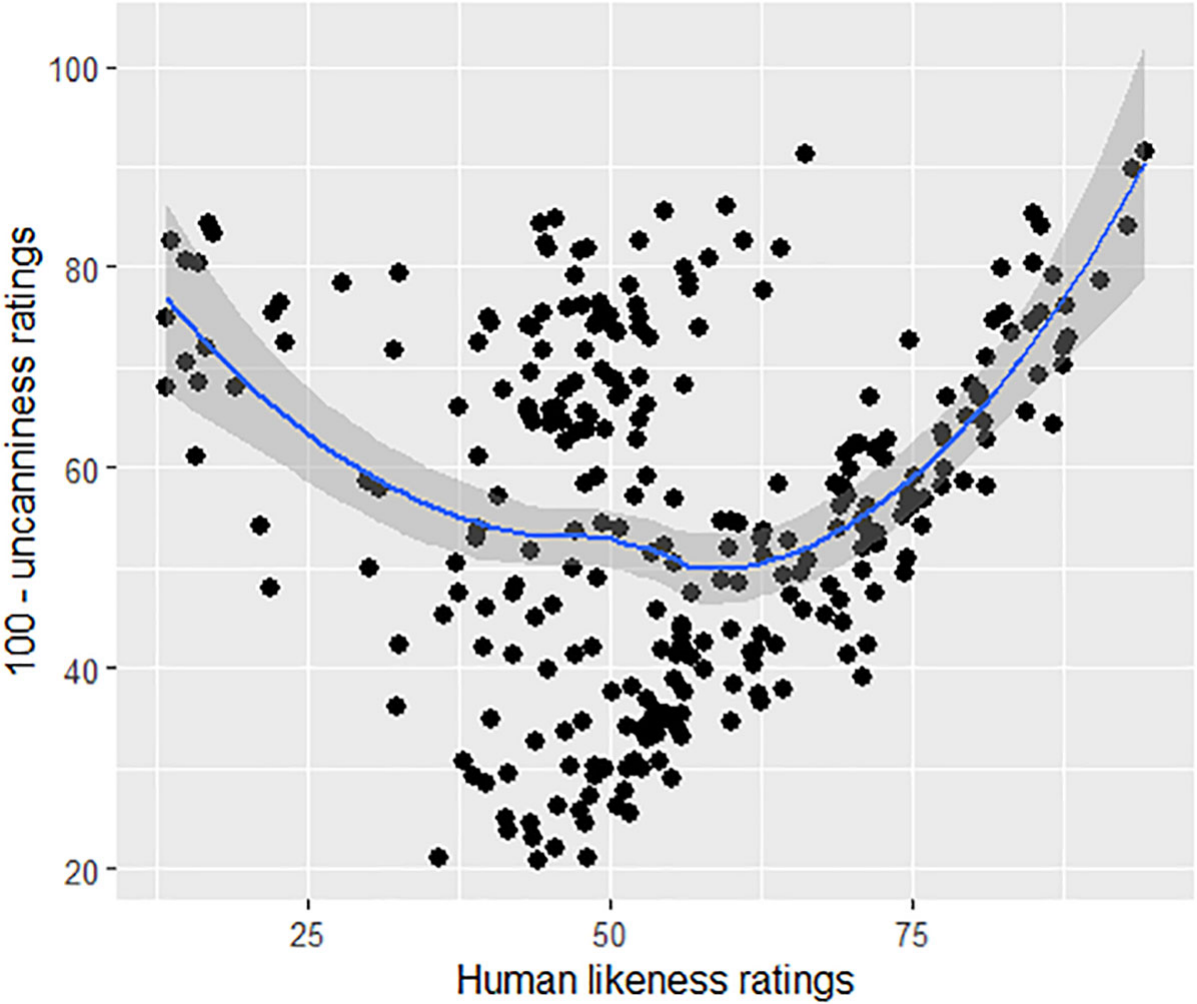
Inverted uncanniness ratings plotted against human likeness ratings. Each point corresponds to a face stimulus per condition, averaged across participants. The blue line represents the regression curve and the grey zone the confidence interval.

To test the first hypothesis, uncanniness ratings were plotted against either linear or quadratic human likeness ratings as fixed effects in a mixed model, including base faces and participants as random effects. Both a linear function (*t*(11570) = 17.45, *p* < 0.001, *R²_corr_* = 0.511), and a quadratic function (*t*(11560) = −30.37, *p* < 0.001, *R²_corr_* = 0.534) of human likeness were significant. The quadratic model was a better fit than the linear model (χ^2^ = 888, *p* < 0.001). The plot is depicted in Figure 8, showing an inverted U-shaped function. Thus, hypothesis 1 was supported.

**Figure 9.**
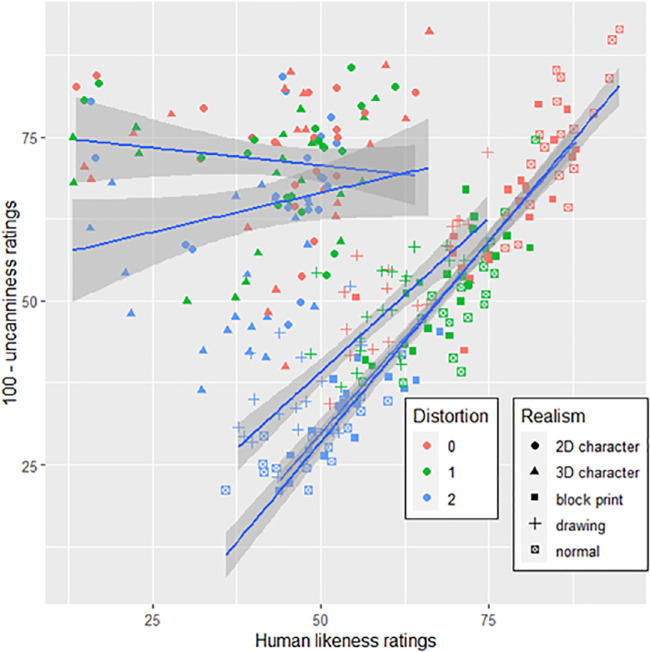
Linear slopes showing the relation between uncanniness and human likeness across faces’ realism levels. The scatterplot is identical to the one in Figure 8, with the addition of depicting distortion levels.

Furthermore, averaged fully realistic faces of all distortion levels ranged in their human likeness ratings from 35.88 to 94.38 and dividing the UV plot across realism levels shows that faces of the first level (fully realistic faces) replicate a curve like the one observed in Experiment 1 (Figure 9). Thus, the data suggest that the range of stimuli used in Experiment 1 corresponds to the rightmost part of the UV curve.

#### Predictors of uncanniness

To test the effects of face realism, familiarity, orientation, and distortion on uncanniness, a linear mixed model was conducted with these predictors as fixed effects and base faces and participants as random effects. Results show significant main effects of realism (*t*(1240) = 7.069, *p* < 0.001), orientation (*t*(11560 = −3.048, *p* = 0.002), familiarity (*t*(44470) = 2.512, *p* = 0.016), and distortion (*t*(10670) = 8.989, *p* < 0.001). Furthermore, significant interactions were found between realism and familiarity (*t*(11240), *p* = −2.514, *p* = 0.012), realism and distortion (*t*(11560) = −4.494, *p* < 0.001), orientation and distortion (*t*(11560) = 4.667, *p* < 0.001), and finally realism, orientation, and distortion (*t*(11560) = −2.304, *p* = 0.0212). No other term was significant (*R²_corr_* = 0.565).

**Figure 10.**
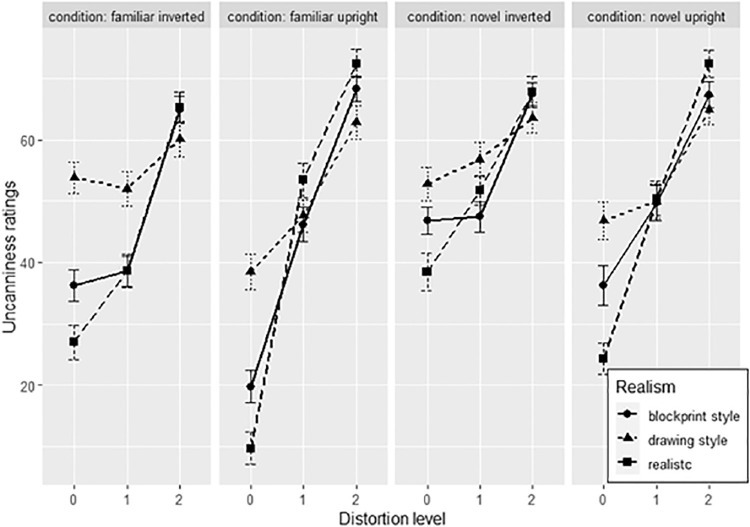
Averaged uncanniness ratings across difference distortion levels (0 = base face), realism levels, and face conditions. Error bars represent standard errors.

**Figure 11.**
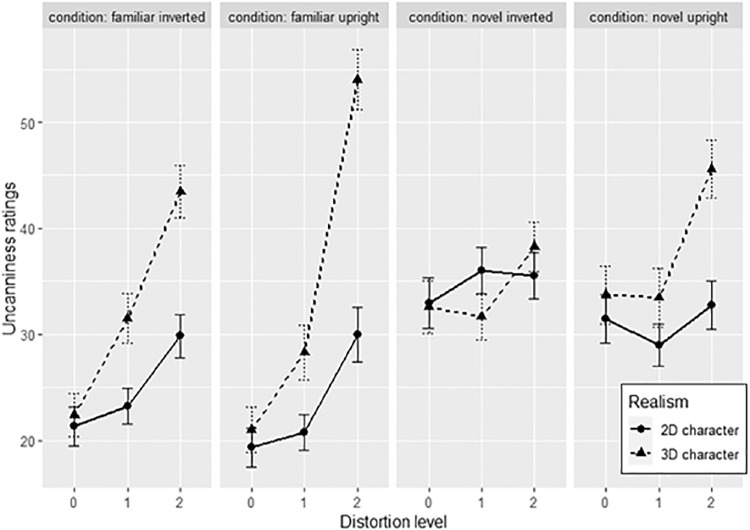
Averaged uncanniness ratings across difference distortion levels (0 = base face), realism levels, and face conditions. Error bars represent standard errors.

In the next sections, results will be analyzed specific to variants of human famous faces and cartoon faces. Data for famous faces (realism levels 1 to 3) are summarized in Figure 10, and data for famous cartoon character faces (realism levels 4 and 5) are summarized in Figure 11.

##### Famous faces

Post hoc Tukey tests were conducted to test the increase of uncanniness between distortion levels 0 to 1 and 1 to 2 for face realism levels and face conditions. For familiar upright faces, distortion significantly increased uncanniness across both distortion levels for fully realistic faces (*t*(2049) = −8.545, *p_adj_* < 0.001 for the 0–1 distortion level; *t*(2049) = −3.451, *p_adj_* = 0.007 for the 1–2 distortion level) and block print style faces (*t*(2049) = −5.099, *p_adj_* < 0.001 for the 0–1 distortion level; *t*(2049) = −4.732, *p_adj_* < 0.001 for the 1–2 distortion difference level), whereas for drawing-style faces, only the 1 to 2 distortion level difference significantly increased uncanniness (*t*(2049) = −3.331, *p_adj_* = 0.011). Thus, for familiar upright faces, distortions increased uncanniness except for slight deviations in highly unrealistic faces.

For familiar inverted faces, all distortions of fully realistic faces increased uncanniness (*t*(2049) = −3.332, *p_adj_* = 0.011 for the 0–1 distortion level; *t*(2049) = −4.862, *p_adj_* < 0.001 for the 1–2 distortion level), but only the 1 to 2 distortion level in block print style faces (*t*(2049) = −6.061, *p_adj_* < 0.001) and none in the drawing-style inverted familiar faces. Thus, the uncanniness sensitivity for familiar inverted faces is decreased when faces are unrealistic.

For novel upright faces, again, uncanniness increased for realistic faces (*t*(2049) = −5.339, *p_adj_* < 0.001 for the 0–1 distortion level; *t*(2049) = −4.191, *p_adj_* < 0.001 for the 1–2 distortion level), but only at the 1 to 2 distortion level for block print style (*t*(2049) = −3.075, *p_adj_* =0.025) and drawing-style (*t*(2049) = −3.132, *p_adj_* = 0.021) faces. Thus, the uncanniness sensitivity for novel faces is decreased for slight deviations if faces are not realistic.

Finally, for novel inverted faces, only the 1 to 2 distortions increase uncanniness for realistic (*t*(2049) = −3.981, *p_adj_* < 0.001) and block print style faces (*t*(2049) = −4.820, *p_adj_* < 0.001). Thus, for novel inverted faces, only strong distortions increase the uncanniness in faces that are either realistic or slightly stylized.

In general, the results show that both familiarity, upright orientation, and high face realism increases the sensitivity of uncanniness to facial distortion. Thus, hypothesis 2 is supported.

##### Cartoon character faces

Furthermore, post hoc Tukey tests were conducted to test the increase of uncanniness across distortion levels 0 to 1, 1 to 2, and 0 to 2 for 3D and 2D cartoon character faces, again across face conditions. For familiar upright faces, uncanniness increased only at the 1 to 2 distortion level for 3D faces (*t*(2122) = −7.723, *p_adj_* < 0.001) and at the 0 to 2 distortion level for 3D (*t*(2122) = −7.339, *p_adj_* < 0.001) and 2D faces (*t*(2122) = −2.987, *p_adj_* = 0.034). Thus, strong deviations were uncanny in both 3D and 2D familiar upright faces.

For familiar inverted faces, uncanniness increased only at higher levels for 3D faces (*t*(2122) = −3.73, *p_adj_* = 0.002 for the 1 to 2 distortion level; *t*(2122) = −5.803, *p_adj_* < 0.001 for the 0 to 2 distortion level). Thus, only stronger deviations in more realistic familiar inverted faces increased uncanniness.

For novel upright faces, only 3D base faces increased in uncanniness at the 0 to 2 distortion levels (*t*(2122) = −3.218, *p_adj_* = 0.016), suggesting that the sensitivity for uncanniness in novel upright faces is only present for strong deviations in more realistic faces.

Finally, uncanniness did not increase across distortion levels on any novel inverted faces. Thus, both novelty and inversion increase the range of acceptable face variation to the point where even strong deviations do not increase uncanniness.

Again, familiarity, upright orientation, and higher face realism increase the sensitivity of uncanniness to facial distortions. Thus, the results support hypothesis 2 even when using “natural” unrealistic base faces.

#### Moderating effect of distortion on base human likeness

Although a quadratic function akin to a UV plot can describe the data, the distribution based on realism level seen in Figure 9 indicates that cartoonish characters can reach levels of human likeness akin to uncanny (deviating yet realistic) stimuli, despite not being uncanny themselves. Whereas the presence of non-uncanny stimuli at the same level of human likeness as uncanny stimuli can be observed in plots in previous research (e.g. Mathur & Reichling, 2016; Pütten & Krämer, 2014), it nevertheless begs the question whether the data can be explained by a function other than a polynomial plot, for example, as indicated in Figure 9, a moderated linear function: If the human likeness of a base face stimulus can be used as a proxy for the closeness to a typical face, it is expected that a higher degree of human likeness also activates a higher degree of configural processing and thus distortion sensitivity, which should reflect in a greater increase of uncanniness across distortion levels for more humanlike base stimuli. Specialized processing would be less important for judging deviations from less humanlike entities (e.g. cartoon faces), and thus deviations would be less increasingly uncanny. Higher face realism would then increase the slope of the effect of distortion on uncanniness, creating a valley-shaped function when plotted across the data. Such a moderating linear function could underlie the relationship between human likeness and uncanniness typically observed as an UV plot, and has thus been investigated in the following exploratory analysis.

A linear mixed model with human likeness, realism, and distortion level as fixed factors, and participant and base face as random factors, was conducted. An interaction between human likeness, realism, and distortion could significantly explain the data (*t*(11520) = −6.185, *p* < 0.001, *R*²_adj_ = 0.55). This interaction model was significantly better at explaining the data that the initial quadratic model (χ^2^ = 968.74, *p* < 0.001). Thus, a linear interaction model between a face's realism level and distortion level across human likeness can better explain uncanniness than the typical quadratic function of human likeness.

### Discussion

#### Uncanny valley and face distortion

The results show that a U-shaped, quadratic function best explained the data, analogous to a U-shaped valley found in previous uncanny valley research (see Diel et al., 2022). Although the UV has been associated with face distortions in past research (Diel & MacDorman, 2021; MacDorman et al., 2009; Mäkäräinen et al., 2014), this study is the first to properly locate face distortions on a UV curve. Results show that distorted version of real faces or stylized variants are located within the UV, compared to undistorted variants to the right and cartoon character faces to the left. Furthermore, the UV observed in this study could be divided into the pre-valley of cartoon faces and valley and post-valley consisting of real face variants, suggesting that an uncanny valley function consists of unrealistic, distant entities (e.g. cartoonish or exaggerated characters, or mechanical and stylized robots) left to the valley, imperfect or distorted variants of realistic human entities at the bottom of the valley, finally followed by fully human entities to the right of the valley. The higher sensitivity for configurations of realistic faces would then explain a harsher judgment toward realistic entities failing to approximate the norm, compared to cartoonish or stylized unrealistic faces.

#### Face realism, familiarity, and orientation

The results show how face realism, familiarity, and orientation interaction with distortion levels to influence uncanniness ratings. Specifically, familiarity and upright orientation increase the sensitivity of uncanniness to facial deviations, which are again more sensitive for more realistic faces. Whereas even subtle deviations could increase the uncanniness in real faces, especially when they were upright and familiar, stronger deviations were needed to increase the uncanniness for stylized faces. Similarly, 2D cartoon faces had a wider range of acceptable, non-uncanny variations than 3D cartoon faces, and for the former, strong distortions only increased the uncanniness when faces were familiar and upright. The results thus indicate that a lower degree of realism generally increases the leeway of face variation, allowing the design of exaggerated facial proportions and expression without risking uncanniness (see also Green et al., 2008; MacDorman et al., 2009; Mäkäräinen et al., 2014). However, familiarity with a cartoon character further narrows the range of acceptable variations, potentially because a deviating familiar face is compared against the much narrower acceptable range of the familiar face representation rather than the acceptable range of all potential facial proportions. Similarly, inversion increases the range of acceptable variations, possibly by decreasing the ability to accurately process subtler configural information and thus potential deviations.

## General discussion

### Uncanniness and deviation from familiarity

Face familiarity increased uncanniness sensitivity. Whereas Mori's (2012) original graph is a good metaphor for possible negative reactions towards artificial humanlike entities, it does not capture some findings in UV research. First, sensitivity of the UV effect towards facial distortions is stronger for more realistic faces compared to less realistic faces (Green et al., 2008; Mäkäräinen et al., 2014). Second, a UV effect has been observed with animal stimuli (e.g. Löffler, Dörrenbacher, & Hassenzahl, 2020; Mitchell, Szerszen, Lu, Schermerhorn, Scheutz, & MacDorman, 2011; Schwind, Leicht, Jäger, Wolf, & Henze, 2018b; Yamada et al., 2013). Third, distortions of the structure of human faces elicits stronger uncanniness ratings than comparable distortions of the structure of cat faces or houses (Diel & MacDorman, 2021). Fourth, as observed in the present study, the sensitivity of uncanniness ratings for distortions is higher for familiar and upright faces compared to novel and inverted faces. Humans usually show a higher level of expertise and special processing for human compared to animal faces (Symons & Roberts, 2006). Furthermore, as face-typical processing is decreased for less realistic avatar faces compared to normal faces (Kätsyri, 2018), a higher level of perceptual experience with a category of faces may increase sensitivity to deviation. Thus, a mechanism underlying the UV effect may be the enhanced ability to detect deviations from familiarized objects and categories, possibly due to an increased experience with recognizing and differentiating individual exemplars. This model would also predict an uncanny valley prevalently for closely human entities with weaker variants for other stimuli like animals and familiar objects. Last, as a topic for future research, manipulating perceptual expertise for a stimulus category should increase the distortion sensitivity of uncanniness ratings.

### Deviation from familiarity and uncanny valley theories

The results are in accordance with theories on the UV resulting from expectation violation (e.g. Saygin et al., 2012), specifically when enhanced by the familiarity with the stimulus category. On the other hand, an increase of sensitivity and uncanniness of configural deviation with increasing familiarity is not predicted by theories relating to threat or disease (MacDorman & Ishgiruro, 2006) or the detection of psychopathic traits (Tinwell et al., 2013). Because the deviations of the familiar faces were not uncanny when the faces were unfamiliar, the configural proportions could not have been related to contagious diseases or potential psychopathic traits. Furthermore, theories relating to categorization ambiguity or difficulty (Cheetham et al., 2011; Yamada et al., 2013) would not necessarily predict that familiarity increases the uncanniness of distorted faces, as an increase of familiarity does move distorted faces closer to the boundary between categories. Nor would it play a role for categorization ambiguity that uncanniness is explained by the sensitivity to detect distortions. Finally, novelty avoidance (Sasaki et al., 2017) would not predict that deviating familiar faces would be more uncanny than the same faces when unfamiliar because the latter would be more novel than the former.

### Rethinking the uncanny valley

An exploratory analysis revealed that the realism level and distortion of a stimulus significantly interact with human likeness to affect its uncanniness. Specifically, the effect of distortion on uncanniness seems to decrease with decreasing human likeness of the base stimulus. This finding urges a rethinking of the current statistical conceptualization of the “uncanny valley” from a single polynomial function of human likeness to a linear function moderated by a third variable. A highly humanlike base stimulus (e.g. a typical human face) may activate specialized processes sensitive to subtle distortions, leading to a stark increase of uncanniness for distorted variants (e.g. distorted faces or android faces). However, less humanlike or realistic base faces (e.g. cartoon faces or mechanical robots) that do not depend on specialized processes are more acceptable of deviations, thus the decrease of likability on the low humanlike part of the UV plot is less steep. In this sense, the UV plot could be better understood by a moderating effect of a stimulus’ specialized processing or distortion sensitivity (which is higher for more humanlike stimuli) on the linear increase of uncanniness due to deviations or distortions of the typical variant.

### Limitations and future directions

The present study provides novel findings on the UV in the context of face familiarity and inversion and the sensitivity to distortions. The following points present possibilities for future directions on this topic.

The tasks in this study used 10 base faces that were presented to the participants multiple times. It is possible that the repeated exposure to base faces increased participants’ short-term familiarity with the faces, which may have confounded the results using familiarity as a predictive factor. Future research could try for more optimal control of face familiarity.

Familiarity is a continuous variable, whereas the present study used a dichotomous coding of familiarity of famous faces. However, participants can vary greatly on their degree of familiarity with different famous faces. Future research can advance the investigation on the effect of familiarity on face distortion processing by using personally familiar faces, or an additional familiarity scale for each base face.

Face realism was only manipulated in Experiment 2, and the decreased uncanniness sensitivity for low-realistic faces has not been associated with general distortion sensitivity. Future research can investigate whether a decrease of face realism also decreases the ability to recognize subtle deviations between faces, which would be expected if a decrease of realism eliminates dimensions within a face space.

Finally, closely familiar faces elicit qualitatively different neurophysiological responses compared to known famous faces (Wiese, Tüttenberg, Ingram, Chan, Gurbuz, Burton, & Young, 2019). As people have more experience with the within-person variability of personally familiar faces, distortion sensitivity to these faces may be even higher. Future research can aim to replicate our findings using personally familiar faces, like those of long-term friends and family members.

## Conclusion

Despite decades of research, not much is yet known about the cognitive mechanisms underlying the UV effect. For example, it is unclear why the UV effect may be stronger for human compared to animal entities, or for more realistic compared to less realistic faces. The present study is the first investigation to show that familiarity with a face increases sensitivity to the UV effect elicited by distortions of facial proportions. The results of this study show that face inversion decreases the sensitivity of uncanniness ratings. Finally, the results of this work indicate that the effects of face familiarity and orientation on “uncanny valley sensitivity” may be mediated by the sensitivity to detect subtle changes in facial distortions. Thus, the present investigation suggests that the degree of perceptual experience with a stimulus category increases the sensitivity to changes within the category, which in turn may increase detection and negative evaluation of distortions. Such a model would explain and predict a stronger UV effect for some stimulus categories, like realistic humans.

## Appendix

**Table A1.** Linear mixed model terms for all models used in study 1 and 2.

Term	*t*-value	Df	Beta estimate	Standard error	*p* value
Distortion, familiarity, and orientation as predictors of uncanniness					
Distortion	29.083	6326	12.828	0.441	0.001
Familiarity	−1.070	6331	−0.927	0.867	0.798
Orientation	−5.345	6326	−4.713	0.882	0.297
Distortion × familiarity	−5.149	6326	−3.134	0.609	0.001
Distortion × orientation	−10.306	6326	−6.429	0.624	0.001
Familiarity × orientation	2.727	6326	3.138	1.217	0.008
All factors	2.292	6326	1.971	0.86	0.010
Distortion, familiarity, and orientation as predictors of “identical” response rates					
Distortion	−65.087	24900	−0.146	.002	<0.001
Familiarity	10.996	34910	0.045	.004	<0.001
Orientation	16.853	24370	0.086	.005	<0.001
Distortion × familiarity	5.419	24900	0.017	.003	<0.001
Distortion × orientation	10.707	24900	0.044	.003	<0.001
Familiarity × orientation	−4.989	24910	0.028	.006	<0.001
All factors	−0.140	24900	<.001	.004	0.888
Distortion, familiarity, orientation, and “identical” response rates as predictors of uncanniness					
Distortion	34.314	9385	12.897	0.3759	<0.001
Familiarity	−6.684	9388	−4.8249	0.7218	<0.001
Orientation	−11.077	9120	−10.2149	0.9222	<0.001
Distortion × familiarity	−4.819	9380	−2.5319	0.5254	<0.001
Distortion × orientation	−11.051	9382	−6.2399	0.5646	<0.001
Familiarity × orientation	7.029	9384	7.5817	1.0787	<0.001
Distortion × familiarity × orientation	3.702	9379	2.8946	0.7819	<0.001
“Identical” response rates only	−38.37	9431	−31.47	0.82	<0.001
